# Salvage Carbon-Ion Radiation Therapy For Locoregionally Recurrent Head and Neck Malignancies

**DOI:** 10.1038/s41598-019-39241-y

**Published:** 2019-03-12

**Authors:** Jing Gao, Jiyi Hu, Xiyin Guan, Jing Yang, Weixu Hu, Lin Kong, Jiade J. Lu

**Affiliations:** 1 Department of Radiation Oncology, Shanghai Proton and Heavy Ion Center, Shanghai, China; 2 Shanghai Engineering Research Center of Proton and Heavy Ion Radiation Therapy, Shanghai, China; 3 Department of Radiation Oncology, Shanghai Proton and Heavy Ion Center, Fudan University Cancer Hospital, Shanghai, China

## Abstract

To investigate the safety and efficacy of salvage carbon-ion radiation therapy (CIRT) in patients with locoregionally recurrent head and neck malignancies. One hundred and forty-one patients with locally recurrent head and neck malignancies previously treated with radiotherapy were salvaged using intensity-modulated carbon-ion radiation therapy (CIRT). The median dose was 60 Gray-Equivalent (GyE) (range 50–69 GyE, 2.0~3.5 GyE/daily fraction). All patients completed planned CIRT except for one. With a median follow-up time of 14.7 (range 1.6–36.4) months, the 1-year overall survival rate was 95.9%. Local, regional, and distant progression free survival rates were 84.9% and 97.7%, and 96%, respectively. Grade 3 or higher acute and late toxicities were observed in 7.1% of the patients. Ten patients developed mucosal necrosis and 4 of these patients deceased. Due to its physical and biological characteristics, CIRT appeared to be an acceptable treatment option for patients with locoregionally recurrent head and neck malignancies after previous radiotherapy. Treatment-induced adverse effects and early response to CIRT were both favorable. Longer follow-up is needed to evaluate the long-term outcome in terms of disease control, survival, as well as potential late effects.

## Introduction

Radiation therapy is one of the major treatment modalities for the management of head and neck malignancies. It is commonly provided to patients diagnosed with locally advanced disease, or with lesions near critical organs for which surgery is not feasible. Despite of the advances in radiation technologies and multidisciplinary approaches, locoregional recurrence remains one of the most commonly observed modes of failure after definitive treatment.

The role of re-irradiation (re-RT) in the management of recurrent head and neck cancer is controversial. When surgical salvage is possible, re-RT is usually deferred because of concerns of serious complications. Recent reports have demonstrated that re-RT is feasible and effective in selected patients using modern RT technologies such as photon-based intensity-modulated radiation therapy (IMRT)^1–4^. However, treatment outcome after re-RT is suboptimal and the reported one-year overall survival (OS) rates ranged between 30–40%.

Charged particles such as proton or carbon-ion have a finite range and a distant Bragg peak^5^. Dosimetry studies have demonstrated that carbon-ion radiation therapy (CIRT) is more precise in the delivery of high-dose radiation to the target volume(s) while sparing OARs as compared to IMRT, thereby enhancing the therapeutic ratio over IMRT in head and neck cancer^6^. CIRT is of particular relevance in the retreatment of head and neck malignancies because the distance between most recurrent tumor target volumes and critical organs at risk (OARs) is usually a matter of millimeters or less. In addition, carbon-ion is a high LET radiation modality that has a relative biologic effectiveness (RBE) ratio of 2~3:1 relative to photon and proton therapy^7^. Therefore, it is reasonable to postulate that this highly precise and biologically effective radiation technique is more effective when used to salvage locally recurrent head and neck malignancies that failed a prior course of high-dose radiation therapy in terms of improved disease control and toxicity profile as compared to IMRT. The effectiveness and potential benefits in the toxicity profile of CIRT in the treatment of locally recurrent head and neck malignancies were only reported in few retrospective series with limited number of patients^8–10^.

At the Shanghai Proton and Heavy Ion Center (SPHIC), CIRT using raster-scanning technology has been used for re-RT for local recurrent head and neck malignancies since early 2015. The aim of this report is to address the early experience in terms of efficacy and toxicity of a relatively large group of patients with locally or locoregionally recurrent head and neck malignancies treated with CIRT using raster-scanning technology.

## Methods and Materials

Between May 2015 and Nov 2017, a total of 156 patients received re-RT for their head and neck malignancies at the Shanghai Proton and Heavy Ion Center (SPHIC). Two patients re-irradiated with proton therapy alone and seven patients treated with proton follow by carbon boost were excluded in this study. Because of their substantial biological differences, 1 patient with skull base meningioma and 5 with skull base or cervical spine chordoma were excluded as well. The remaining 141 patients were treated for local or locoregionally recurrent head and neck tumors including: un-/poorly- differentiated or keratinized head and neck squamous cell carcinoma (HNSCC, n = 110), adenoid cystic carcinoma (ACC, n = 10), and mucoepidermoid carcinoma (n = 3), adenocarcinoma (n = 3), spindle cell sarcoma (n = 1) osteosarcoma (n = 1), rhabdomyosarcoma (n = 2), pleomorphic sarcoma (n = 1), parotid mixed tumor(n = 1), primitive neuroectodermal tumors PNET (n = 1), and radiation-induced secondary primary malignancy (n = 8). All recurrences were diagnosed by imaging studies and/or pathological confirmation.

This is an observational study and no experiment using human or animal sample(s) was involved. Patients in this cohort were treated according to our institutional research protocols that were approved by the institutional review board (IRB) of the SPHIC. All protocols were carried out in accordance with the ethical standards laid down in the 1964 Declaration of Helsinki and its later amendments. Informed consents were obtained from all patients treated on the protocols.

### Previous radiation therapy

All patients had been treated with one and only one previous course of photon-based radiation therapy and recurred >11 months after the completion of the initial radiation. One hundred and twenty-nine (129) patients received fractionated photon radiotherapy including IMRT. One (1) patients of radiation induced skull base small-round cell malignant tumor had Gamma Knife radiosurgery. Information on previous radiotherapy for the rest 11 patients was unknown.

The characteristics of the patients, their malignancies, initial treatments, and any therapy for recurrence prior to re-RT are summarized in Table 1.

**Table 1:** Characteristics of patients, disease, and initial treatment.

Characteristics	n	%
**Age**		
≥50	67	47.5
<50	74	52.5
median	49	
range	17–82	
**Gender**		
male	101	71.6
female	40	28.4
**Tumor sites**		
Nasopharynx	114	78.1
Nasal cavity or paranasal sinuses	12	8.2
Oropharynx	5	3.4
Salivary glands	4	2.7
Skull base	1	0.7
Larynx and hypopharynx	2	1.4
Other	3	2.1
**Histology**		
Squamous cell carcinoma (including poorly or un-differentiated)	110	75.3
Adenoid cystic carcinoma	10	6.8
Mucoepidermoid carcinoma	3	2.1
Adenocarcinoma	3	2.1
Spindle cell sarcoma	1	0.7
Osteosarcoma	1	0.7
Rhabdomyosarcoma	2	1.4
Pleomorphic sarcoma	1	0.7
Parotid mixed tumor	1	0.7
Primitive neuroectodermal tumor	1	0.7
Radiation induced secondary primary malignancy	8	5.5
Recurrent T stage*		
rT1	19	13.6
rT2	15	10.7
rT3	41	29.3
rT4	52	37.1
rT0 (+retropharyngeal node)	13	9.3
Recurrence clinical stage*		
I	18	12.9
II	25	17.9
III	41	29.3
IVA/B	56	40.0
**Time to recurrence**		
≥3 years	69	48.9
<3 years	72	51.1
median (mo)	36	
range (mo)	11–257	
**Original RT technique**		
IMRT	129	91.5
Stereotactic radiosurgery	1	0.7
Not recorded	11	7.8
**Pre-Salvage-PT therapy**		
Surgery	23	16.3
Chemotherapy	64	45.4
None	54	38.3

RT-radiotherapy; PT-particle radiotherapy; CIRT-intensity modulated carbon-ion radiotherapy.

*AJCC staging system for musculoskeletal tumors was used for chordoma staging. Middle ear cancer did not staged with the AJCC staging system.

### Pretreatment evaluations

Required pretreatment examinations consisted of a thorough history and physical examination, complete blood counts, serum electrolyte tests, liver and renal function tests, electrocardiogram, urinalysis, and pregnancy test for female patients of child-bearing age. Magnetic resonance imaging (MRI) of the head and neck are mandatory unless clinically contraindicated for all patients to evaluate the extent of the disease. Whole body PET-CT and endoscopy examinations were performed if clinically indicated. Patients who present with regional lymphadenopathy were required to be evaluated for neck dissection.

### Intensity-modulated carbon ion radiation therapy

All patients were re-irradiated using CIRT with raster beam scanning technology as published previously^11^. Briefly, patients were typically immobilized in the supine position with thermoplastic masks. CT simulation without contrast from the vertex to the inferior margin of clavicular heads was performed with 1 mm slices. MRI-CT fusion was performed for all patients for target volume delineation. The gross tumor volume (GTV) was contoured as the macroscopic lesion seen on CT, PET-CT, and MRI. The clinical target volumes (CTVs) included the GTV as well as a margin of 3–5 mm to account for potential microscopic spread. Smaller margins of CTV were allowed for lesions close to critical OARs that were previously irradiated. Prophylactic irradiation for subclinical disease to any uninvolved regions regardless of the probability of disease involvement was not administered. An additional 1–3 mm margin was added to the CTV to create the planning target volume (PTV) to allow for setup variability and uncertainty in dose distribution.

OARs required for all patients were defined according to the following priority: brainstem, spinal cord, optic nerves/chiasm, temporal lobes, pituitary gland, eyes (including lens), temporomandibular joints, and parotid glands. A 70% dose recovery from the dose of the initial radiation therapy course was used for all patients as their dose recurred >12 months after the completion of the initial radiation, based on the radiobiological conclusions of Nieder *et al*.^12^. The dose constraints of the OARs are based on TD5/5 described by Emami except for optic nerve (D_20_ < 30GyE), brain stem (D_max < _45 GyE), spinal cord (D_max < _30 GyE), and temporal lobes (V_40_ < 7.66cc; V_50_ < 4.66cc) which were based on previous experience from the National Institute or Radiation Science (NIRS) of Japan^13,14^.

Treatment planning was performed using biologic treatment plan optimization using the Syngo^®^ treatment planning software system (version VC 11) which takes into account local values of the RBE calculated by the TRiP software based on the local effect model (LEM). An alpha/beta value of 2 was used for normal tissues.

**Figure 1:**
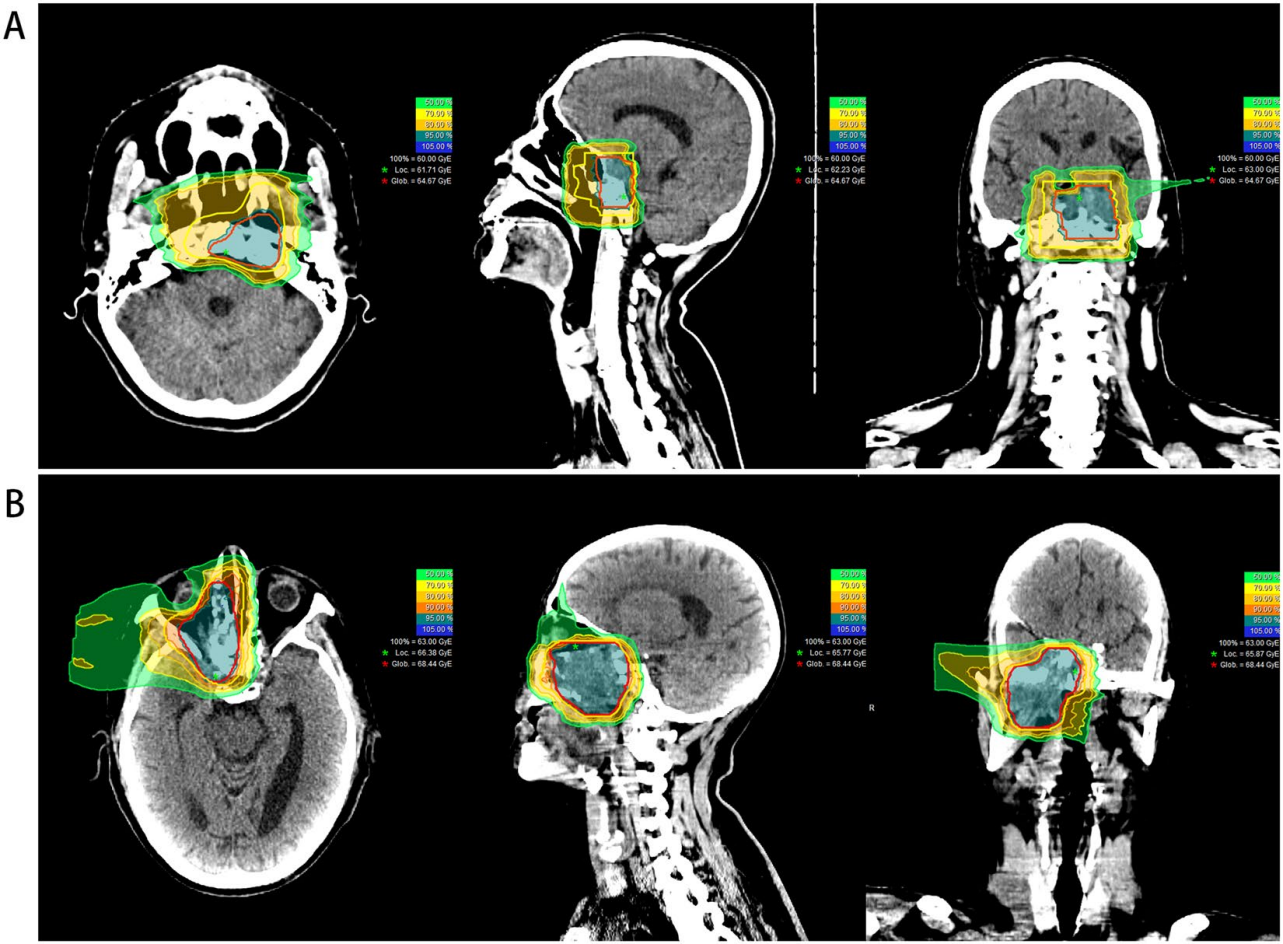
Typical CRIT treatment plans for patients with skull base recurrence. (**A**) A patient with rT1N0M0 locally recurrent small round cell tumor previously treated with surgery and stereotactic radiosurgery; (**B**). A patient with rT4N0M0 locally recurrent NPC previously treated with IMRT.

In most patients, 2–3 treatment portals were used for plan delivery from the primary horizontal beam. The treatment plans of a patient with locally recurrent base of skull small round cell tumor and another with locally recurrent nasopharyngeal cancer are illustrated in Fig. 1A,B.

The median dose used for patients who received CIRT alone was 60 Gray-Equivalent (GyE) (range 50–69 GyE, 2.0–3.5 GyE/daily fraction) excluding one patient who discontinued CIRT after 4 fractions due to rupture of an intercurrent optic artery aneurysm.

Prior to each treatment, patient positioning was evaluated and corrected using orthogonal X-rays. Weekly evaluations were required for all patients for potential adverse events during CIRT. Weekly CT scans of the head and neck were performed after the second week of CIRT to ensure the consistency of body contour and dose distribution. Re-planning was utilized if clinically indicated.

### Chemotherapy

Induction chemotherapy was encouraged but not mandatory for all patients with locally advanced recurrent HNSCC, except for patients accrued for clinical trials on combined chemoradiotherapy. Concurrent chemotherapy was not recommended during CIRT except for patients participating clinical trials that required such treatment.

### Follow-up evaluations

According to our institutional “Standard Operation Protocol for Follow-up after CIRT”, all patients were required to be followed up by their primary radiation oncologists. The first follow-up visit was scheduled within 6 weeks after completion of treatment, thereafter in 3 months intervals for the first 2 years, every 6 months in the following 3 years, then annually indefinitely. Thorough clinical assessments including endoscopic procedures (if indicated) were required at each follow-up. Contrast-enhanced MRI-examinations of the head and neck were required at every follow-up session with the first scan scheduled at the last week of CIRT. PET-CT scans for assessment of local or metastatic failure were optional and were ordered at the discretion of the radiation oncologist. Endocrinological check-up examinations were ordered if clinically indicated for patients re-irradiated to the skull-base region.

### Data analysis

Clinical response to salvage CIRT was based on the results of post-treatment MRI and/or PET-CT scans and physical examination using the RECIST criteria (version 1.1)^15^. The duration of OS time was calculated from the diagnosis of recurrence until death or the date of the last follow-up session for patients still alive. The duration of time to local, regional, or distant failure was measured from the date of the diagnosis of recurrence until documented failure. Imaging confirmation of local, regional, and/or distant recurrence or progression were required after salvage CIRT to define disease progression and to calculate progression-free survival. The cumulative incidences of local and regional failure were calculated, respectively, with death treated as a competing risk. Local failure refers to the recurrences within or at the margin of the CIRT fields and regional recurrence refers to the recurrences in the regional draining lymph nodes only. Overall, disease-specific, and distant metastasis-free survival rates were calculated using the Kaplan-Meier method. All statistical analyses were performed using R (version 3.4.3). Acute and late toxicities, which occurred within or after 3 months after the completion of CIRT, were measured using the CTC AE (version 4.03).

### Statement of ethical standards

This is an observational study and no experiment using human or animal sample(s) was involved. Some patients in this cohort were treated according to our institutional research protocols, and IRB approvals were obtained for all research protocols involved in this report. All protocols were carried out in accordance with the ethical standards laid down in the 1964 Declaration of Helsinki and its later amendments. Informed consents were obtained from all patients treated on the protocols.

## Results

At the time of analysis, the median follow-up time was 14.7 months (range 1.6–36.4 months) for the entire cohort of patients. All except one patient completed planned re-irradiation using CIRT without any interruptions for any reason including treatment-induced adverse events. One patient discontinued CIRT after 4 fractions due to hemorrhage from rupturing of an intercurrent optic artery aneurysm.

### Local and regional control

**Figure 2:**
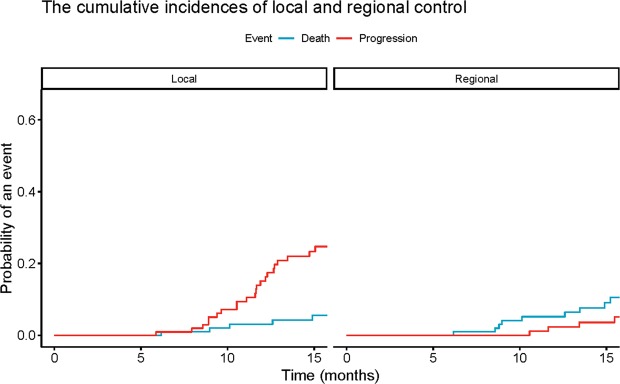
Cumulative incidence of local and regional failure with death as a competing risk.

Local and or regional failure occurred in 39 patients, including 37 local failures within or at the margin of the CIRT fields and 2 regional recurrences in the regional lymph nodes. The median time to locoregional recurrence was 12.9 months (range 5.9–31.1 months). The 12-month incidence of local and regional control with death as a competing risk was 84.9% and 97.7%, respectively (Fig. 2).

### Freedom from distant metastasis

**Figure 3:**
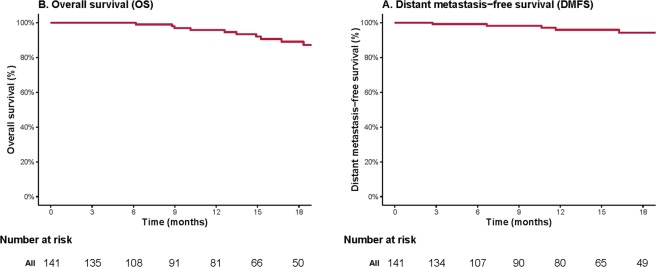
The actuarial distant metastasis-free (**A**) and overall survival rates (**B**).

Six (6) patients developed distant failure including 4 with bone (the most common site) metastases during their follow-up, with a median time of 11.2 (range 2.7–24.4) months. The actuarial 12-month distant metastatic-free survival (DMFS) rate was 96% (95%CI:92–100%) (Fig. 3A).

### Overall and disease specific survival

Twelve patients died at a median of 14.2 (range 6.2–20.5) months. The 12-month overall survival (OS) and disease-specific survival (DSS) rates were both 95.9% (95% CI: 92–99.9%) (Fig. 3B).

### Acute and Late Adverse Effects

Commonly observed treatment-induced adverse effects included Grade 1 or 2 local alopecia, mucositis with dysphagia, and skin erythema (Table 2). Grade 4 acute hemorrhage was observed in two patients during treatment, including one case of confirmed rupturing of an inter-concurrent optic artery aneurysm unrelated to the disease or treatment, and another case was probably associated to patient’s re-irradiation using CIRT. Both patients had radiation-induced second primary tumors.

**Table 2:** Acute and Subacute Adverse Effects.

	Grade 1–2	Grade 3–5
Dermatitis	10 (7.1%)	0
Mucositis	26 (18.4%)	0
Xerostomia	5 (3.5%)	0
Nausea	2 (1.4%)	0
Headache	1 (0.7%)	0
Hemorrhage	0	1* (0.7%)

*Excluded 1 case of hemorrhage due to confirmed rupturing of an inter-concurrent optic artery aneurysm unrelated to the disease or treatment confirmed on angiogram.

Ten patients developed mucosal necrosis after three months of follow up. Four of the 10 patients died due to hemorrhage. Eight patients develop asymptomatic temporal lobe necrosis and one patient presented with amnesia. Three patients required G-Tube placement because of dysphagia caused by cranial neuropathy. Profiles of the late toxicities were detailed in Table 3.

**Table 3:** Type and frequency of late toxicities.

	Grade 1or 2	Grade 3 or higher
Xerostomia	1 (0.7%)	1 (0.7%)
Mucosal necrosis	0	10 (7.1%) *
Temporal lobe necrosis	8 (5.7%)	1 (0.7%)
Hearing loss	1 (0.7%)	0
Cranial neuropathy	1 (0.7%)	3 (2.1%)

*Including 4 died of hemorrhage secondary of mucosal necrosis.

## Discussion

In this study, we analyzed the early outcomes of 141 patients re-irradiated using CIRT for their locoregionally recurrent head and neck malignancies after previous high-dose radiation therapy. This represents the largest, single-institutional series of re-irradiated head and neck malignancies and the largest overall series that reports the outcomes with carbon-ion radiotherapy as the principal re-RT modality. At the time of this analysis, 12 patients had deceased including 4 from disease progression, 4 from late toxicities (i.e., massive hemorrhage), and 4 from unrelated inter-concurrent diseases. With a median follow-up time of 14.7 (range 1.6–36.4) months. The 12-month OS and DSS rates were both 95.9%. Only two patients experienced acute Grade 4 hemorrhage during their treatment, including one from a confirmed ruptured optic artery aneurysm unrelated to disease or treatment. And severe late adverse effects occurred in nearly 10% of patients.

Our preliminary findings suggest that re-RT with CIRT at a median dose of 60 GyE (range 50~69 GyE at 2.0~3.5 GyE/daily fraction) is feasible and potentially effective for controlling locally recurrent foci of certain types of head and neck malignancies. Despite the relatively short median follow-up time of 14.7 months, our outcomes, including the short-term survival data and paucity of SAEs, are encouraging as compared to those after re-RT with conventional technique or IMRT. Re-RT with conventional radiotherapy or IMRT for head and neck tumor recurrences is limited by doses delivered to brainstem, brain (temporal lobes), cranial nerves especially the optic nerve and chiasm, as well as pharyngeal mucosa. As such, disease control could be hampered and severe toxicities could be severe. In a study of 84 patients re-treated with IMRT with or without chemotherapy, Duprez *et al*. found that the long-term LC and OS rates were only 40% and 20%, respectively, after a median follow-up of 19.8 months. In addition, close to 40% of patients developed Grade 3 acute and late toxicity^16^. In a larger series of 105 patients re-irradiated for locally recurrent head and neck cancer, only 18 patients were alive with disease free with a median follow up of 35 months. The 2-year local recurrence free survival was 52% for patients treated with IMRT, which is significantly better than the 20% observed for those treated with conventional techniques. Severe toxicities (Grade 3 and 4 combined) were reported in close to 40% of those patients, and the median time to the development of Grade 4 late toxicities was 6 months after re-irradiation^1^. A more recently published series of 505 locoregionally recurrent head and neck cancer patients salvaged with IMRT from 9 academic institutions in the United States revealed that 22.1% and 16.7% of patients experienced severe (≥Grade 3) acute or late toxicities, respectively^17^. The 2-year OS rate was 49.3% for patients received salvage radiation dose of ≥66 Gy versus a dismal <35% for those received <66 Gy.

It is important to limit the re-RT dose to the disease foci only without overdosing the normal surrounding tissues. The use of novel radiation techniques, such as proton or CIRT, in combination with advanced imaging to identify locally recurrent disease could improve overall outcomes. Proton or carbon-ion radiation provides advantageous physical characteristics such as a sharp lateral penumbra, low energy deposition within the entry path prior to and after the Bragg peak formed by the steep dose deposition, thus possessing a dose delivery with a finite range. With these physical characteristics, the commonly observed severe adverse effects mentioned above after conventional radiation or IMRT might be reduced through improved sparing of normal tissue.

This is especially important in re-RT of head and neck area for patients who have completed a previous course of high-dose radiation. A number of studies have reported improved dose distributions using particle therapy for primary or recurrent head and neck tumors with acceptable clinical outcomes^10,18^. In addition to its improved physical characteristics, high LET radiation such as CIRT has a significantly higher relative biological effectiveness (RBE) as compared to those of photon and proton radiation. The high LET CIRT induces more damage in the form of direct DNA double strand breaks, which is more difficult to repair^19^. As such, improved clinical results could be expected if CIRT is used as a re-irradiation modality especially for the photon-resistant tumor cells.

Our outcomes are highly comparable to patients with base of skull recurrences treated with similar technology and facility at the Heidelberg Ion Therapy center (HIT). In a recently published paper on reirradiation with CIRT for 18 patients with recurrent skull base diseases, the median dose of 51 GyE (over 17 daily fractions, 7 days a week) were well tolerated^8^. No patient experienced moderate or severe acute or late side effects. Grade 1 or 2 early or late toxicity were only observed in 5 patients with a median follow up of 2 years. Results of other reports on re-irradiation using CIRT (median dose = 51 GyE) for head and neck recurrences revealed similar finding. In 52 patients retreated for recurrent ACC treated with CIRT, Jensen *et al*. achieved a local control of 70% with a median follow-up of 14 months^20^. The researchers observed no Grade 3 toxicities, although the treatment volume was extensive in a few patients included. In a more recently published paper, Romesser *et al*. reported the results of 92 patients with locoregional recurrent head and neck cancer re-irradiated with proton beam radiation^21^. The 1-year OS, locoregional control, and DMFS rates were 65.1%, 74.9%, and 84%, respectively. Furthermore, Grade 3 acute mucositis, esophagitis, and dysphagia each occurred in approximately 10% of patients. Another 3.3% of patients experienced Grade 3 dermatitis. Late Grade 3/4 dysphagia and dermatitis occurred in 7.1% and 8.6% of patients, respectively. Two patients (2.9%) died of hemorrhage as a Grade 5 event. Similar findings were presented in the 60 patients salvaged at the M.D. Anderson Cancer Center (MDACC) and the 61 patients re-irradiated at the University of Indiana (UI) using proton radiotherapy^22,23^. The 1-year rates of locoregional failure-free and overall survival were 68.4% and 83.8% respectively as reported from MDACC. And the 2-year rates of OS, locoregional failure reported from the Indiana University, with a median follow-up time of 15.2 months, were 32.7% and 19.7%. However, 38.3% of patients developed distant metastasis. Severe (Grade 3~5) acute adverse effects were seen in 30% and 14.7%, and severe late adverse effects were observed in 20% and 24.6% of respectively, in both studies.

It seems that patients salvaged with CIRT had better outcomes in terms of 1-year OS and locoregional disease control as compared to those treated with proton therapy. However, it must be emphasized that the pathology and severity of the conditions were different. For example, patients in our series failed after only one course of previous radiation, but close to 18% of the 92 patients salvaged with proton radiotherapy reported by Romesser *et al*. experienced 2 or more courses of radiotherapy. Furthermore, 16 patients had more indolent cancers of the salivary glands in our series. Nevertheless, the low incidence of severe (≥Grade 3) acute or late toxicities from particle radiation therapy seemed to be consistent among all published literatures.

We are not able to explain the underlying reason for such a low incidence of acute and sub-acute toxicities observed in patients received CIRT in our institution and HIT. A recently published studied the effects of ultra-high dose-rate radiation therapy in normal and cancer tissues, and reported that radiation delivered in less 500 ms could spare normal lung tissue while the efficacy to cancer tissue remains unchanged^24^. The scanning speed of the IONTRIS system for carbon-ion beam is 100 ms/slice. We hypothesize that the high scanning speed of CIRT may have played a role in limiting the acute adverse effects in our patients, and this topic will be one of our future research foci in our institution.

Despite of the absence of moderate or severe short-term side effects, the most concerning limiting factor for the use of re-irradiation with any beam type is late effects of the vital organs. Of all the critical OARs, brain stem reaction is of paramount importance in patients of head/neck and base of skull tumors. Conventional radiation techniques did not allow for sparing of dose-limiting organs such as spinal cord and brainstem. A number of organs such as heart, kidney, and bladder do not recover but the spinal cord and brainstem may recover from irradiation damage. In a study evaluating recovery from radiation injury to the spinal cord, Ang *et al*. demonstrated that the spinal cord of rhesus monkey recovers within 1–3 years from previous radiation dose of 45 Gy, and >90% recovery could be achieved after three years. Re-irradiation up to 68 Gy in conventional fractionation was well tolerated^25^. Nevertheless, one must remember that severe adverse effects such as injury to the cranial nerves, spinal cord or brainstem, once developed, are devastating and debilitating. The signs and symptoms may occur at any time in the course of follow-up. In an early study on radiation injury to cranial nerves, we have demonstrated that cranial nerve palsy may occur up to 34 years after the completion of radiation therapy^26^. Tolerance of the brainstem and cervical spinal cord to radiation therapy has also been addressed clinically in patients treated with proton radiation therapy. In a large series of 367 patients treated for skull base tumors using photon and protons, less than 5% of patients (17 of 367) developed treatment-induced adverse effects, with subsequent death in three patients. The risk for severe adverse-effects was not only associated with the dose to the brainstem but also with the volume of brainstem irradiated to high doses. Radiation doses of 60 GyE or higher is a significant predictive factor for brainstem damage in multivariate analysis^27^. However, tolerance of the spinal cord to CIRT has not been evaluated in clinical settings.

In the present report we have demonstrated that CIRT is potentially effective in the re-treatment of recurrent tumors in the head and neck. Our study has a number of pitfalls. First, although we presented the largest series of locoregionally recurrent head and neck malignancies salvaged with re-irradiation using particle therapy, the number of patients of individual categories are limited and heterogeneous in-terms of disease type, size of the tumor, and modalities used for previous radiation therapy. Direct comparison of the treatment outcome such as OS between different diseases cannot be performed due to the differences in prognosis for different pathologies. In addition, the follow-up time is relatively short for a number of more indolent diseases such as recurrent ACC. Third, although most patients received CIRT alone for salvage treatment, dose and fractionation schemes used at different periods of time were heterogenous in our series. Therefore, no clear dose–response association can be revealed by our results. Further, published data on the tolerance of OARs for CIRT is scant. Defining proper tolerance doses is especially challenging for salvage treatment as previous irradiation usually had heterogenous dose/fraction and latent period before relapse. Under such scenario, we used the TD5/5 proposed by Emami *et al*. instead of QUANTEC reports for convenience, together with few limiting-doses of CIRT suggested by NIRS^28^. Clearly, further investigation, preferable in prospective fashion, with larger sample sizes, will be needed. Nevertheless, our results strongly support the use of CIRT for retreatment of patients with recurrent tumors in the head and neck, considering the relatively favorable survival and disease control data as compared to those from historical literature for this highly challenging condition. On a side note, the dose-volume data for all RT-naive patient treated with CIRT at SPHIC are being collected prospectively with an aim to define tolerance dose once sufficient follow-ups are achieved.

Accurate delivery of CIRT requires not only proper planning but also precise daily positioning of the patients. Unlike in photon-based IMRT, most particle therapy centers use orthogonal imaging for position verification. The use of cone-beam CT (CBCT) in daily setup is an interesting topic. Change of tumor positions relative to other structures especially bones pose a dilemma: if position is corrected based on the tumor position on CBCT, the dose coverage to CTV may be skewed more substantially from the plan than without such correction, if the density of the structures (such as bone) in the beam path altered. Such phenomenon is not substantial in photon radiation. As such, the value of CBCT in daily treatment is only in verifying rather than guiding the positioning at this time. The effective use of CBCT in particle radiotherapy optimally depend on real-time re-planning, a hot topic under active investigation which requires the development of ultra-fast plan calculation. Our institute is currently installing a CT on rail for daily verification and potentially real time re-planning, which will be commissioned in 2019.

## Conclusion

At the doses/fractionation range used in our cohort of 141 patients, only one patient experienced a treatment-induced acute SAE. Severe late toxicities occurred in approximately 10% of patients. The 1-year OS and LC rates of 95.9% and 84.9% support the use of CIRT for patients with locoregionally recurrent tumors after previous radiation therapy. However, longer follow-up is needed to evaluate the definitive efficacy and late toxicity profile of CIRT in the re-RT of head and neck malignancies. Further investigations preferably in prospective fashion are needed to understand the optimal dose for different types of diseases.
